# A case of successful management for spontaneous rupture of paraganglioma treated with preoperative transcatheter arterial embolization

**DOI:** 10.1186/s40792-024-01907-9

**Published:** 2024-06-21

**Authors:** Masataka Nakagawa, Naoki Tanimine, Hiroshi Sakai, Ryosuke Nakano, Shintaro Kuroda, Masahiro Ohira, Hiroyuki Tahara, Kentaro Ide, Tsuyoshi Kobayashi, Kouji Arihiro, Hideki Ohdan

**Affiliations:** 1 Department of Gastroenterological and Transplant Surgery, Hiroshima University Hospital, 1-2-3 Kasumi, Minami-Ku, Hiroshima, 734-8551 Japan; 2 Department of Pathology, Hiroshima University Hospital, 1-2-3 Kasumi, Minami-ku, Hiroshima, 734-8551 Japan

**Keywords:** Paragangliomas, Pheochromocytomas and paragangliomas, Pheochromocytoma, Spontaneous rupture of paraganglioma, Transcatheter arterial embolization

## Abstract

**Background:**

Tumors arising from catecholamine-producing chromophil cells in paraganglia are termed paragangliomas (PGLs), which biologically resemble pheochromocytomas (PCCs) that arise from the adrenal glands. Spontaneous rupture of a PGL is rare and can be fatal. Although elective surgery for ruptured PCCs after transcatheter arterial embolization (TAE) has been shown to provide good outcomes, the efficacy of TAE pretreatment for ruptured PGL remains unknown.

**Case presentation:**

A 65-year-old female with hypertension and tachycardia was diagnosed with a 3-cm PGL located behind the inferior vena cava. The patient was scheduled to undergo an elective surgery with antihypertensive therapy. However, she presented with a chief complaint of abdominal pain and was diagnosed with intratumoral hemorrhage. Urgent TAE was performed that successfully achieved hemorrhage control. After TAE, serum levels of both epinephrine and norepinephrine were within the normal range. Abdominal computed tomography revealed resolving retroperitoneal hematoma. Elective open surgery was performed without significant intraoperative bleeding or fluctuations in blood pressure.

**Conclusion:**

We report a case of successful preoperative TAE for functional PGL to control intraoperative blood pressure fluctuations and bleeding. Preoperative TAE could be a useful procedure for the surgical preparation of functional PGL, including unruptured cases.

## Background

Tumors arising from catecholamine-producing chromophil cells in the paraganglia are called paragangliomas (PGLs) and are biologically similar to tumors arising from the adrenal medulla, termed pheochromocytomas (PCCs). Owing to their similarities, PCC and PGL are collectively referred to as pheochromocytomas and paragangliomas (PPGL) [1].

Functional PPGL is a potentially malignant neuroendocrine tumor that presents with various clinical symptoms such as hypertension, headache, palpitations, and sweating. Although the first-line treatment for functional PPGL is surgical resection preceded by antihypertensive therapy using alpha-1 blockade, surgical resection carries the risk of perioperative morbidity and mortality due to hypercatecholamine crisis [2, 3]. Spontaneous rupture of a PPGL, which is rare in the natural course of the tumor, could lead to a hypercatecholamine crisis. Previous cases of ruptured PCC showed that elective surgery after appropriate management, such as blood pressure control and transcatheter arterial embolization (TAE), can lead to good outcomes [3, 4]. Reports of PGL treated with TAE are rare, and only one case report was found in our best effort [5]. Herein, we report a successfully managed case of spontaneous PGL rupture in a 65-year-old female who underwent preoperative TAE for perioperative hemostasis with elective resection.

## Case presentation

**Fig. 1:**
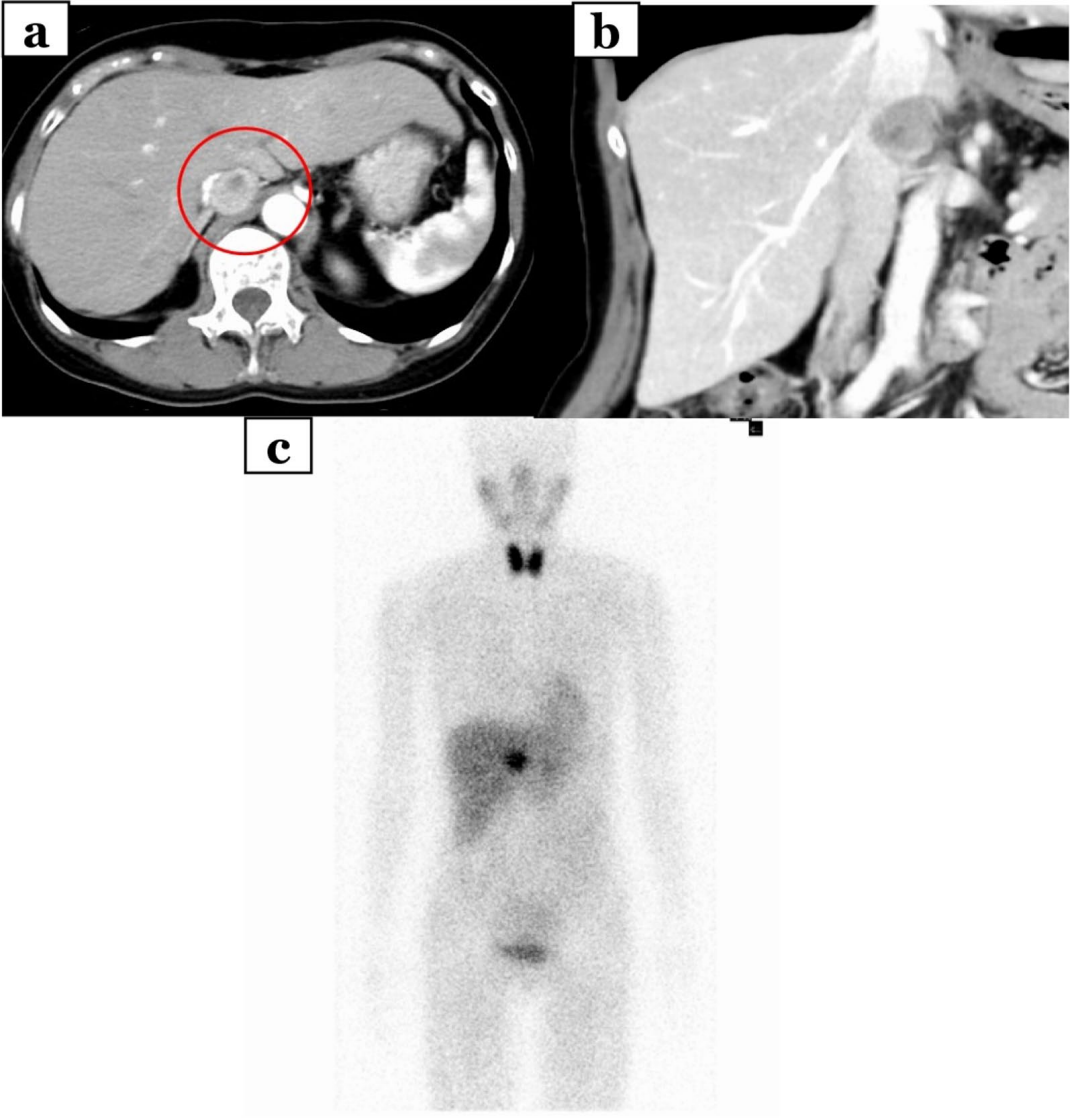
Image findings for diagnosis of paraganglioma. **a**, **b** Before emergent admission, computed tomography (CT) showed a well-defined 30-mm tumor on the dorsal aspect of the inferior vena cava. **c** 123I-metaiodobenzylguanidine scintigraphy shows abnormal hyperaccumulation in the tumor area

**Fig. 2:**
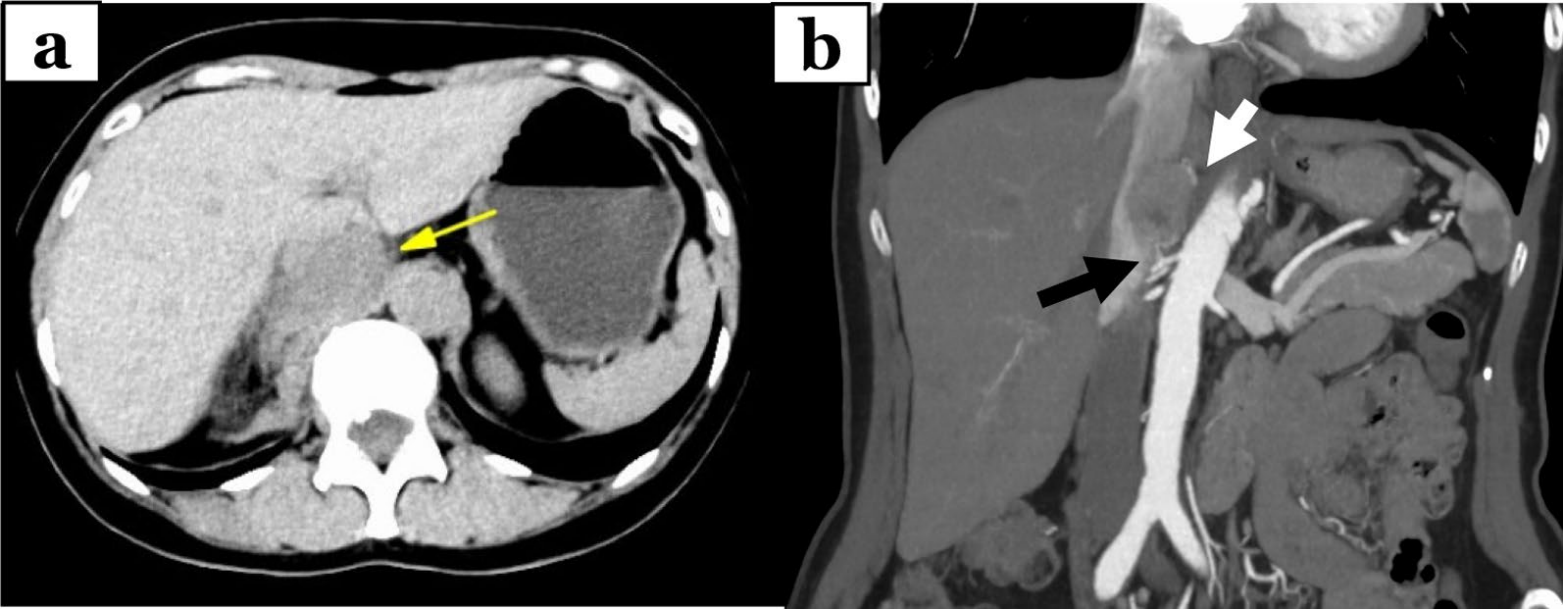
Computed tomography findings at the time of emergent admission. **a** Computed tomography (CT) showed an enlarged mass and surrounding retroperitoneal hemorrhage. **b** CT showed that the tumor was dominated by two arteries

**Fig. 3:**
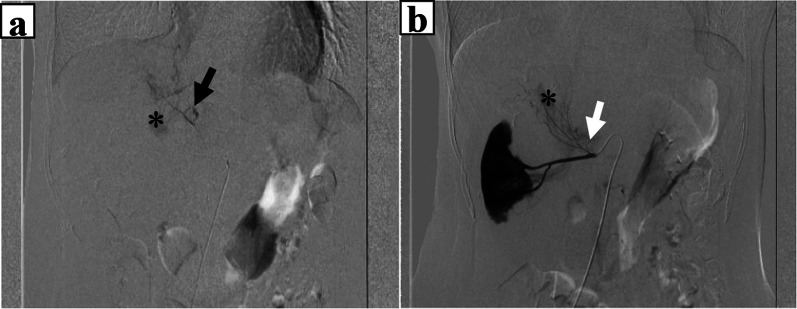
Emergent angiography findings for retroperitoneal hemorrhage. Angiography examination confirmed an extravasation at the tumor site (*). The blood flow was supplied by **a** the right inferior transverse artery (black arrow), which branched directly from the aorta and **b** the right adrenal artery (white arrow), which originated in the right renal artery

**Fig. 4:**
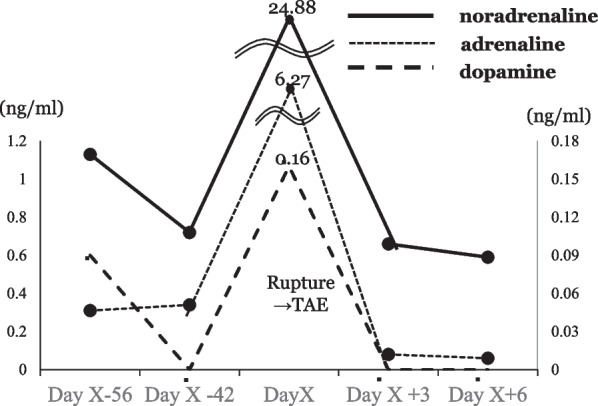
Kinetics of serum catecholamines in clinical course. The patient was diagnosed as PGL 56 days before rupture event. The line chart shows serum level of catecholamines before and after transcatheter arterial embolization (TAE, day X). The levels of noradrenaline (black line), adrenaline (dotted line), and dopamine (dashed line) showed a surge at the time of tumor rupture and normalized rapidly after TAE treatment

**Fig. 5:**
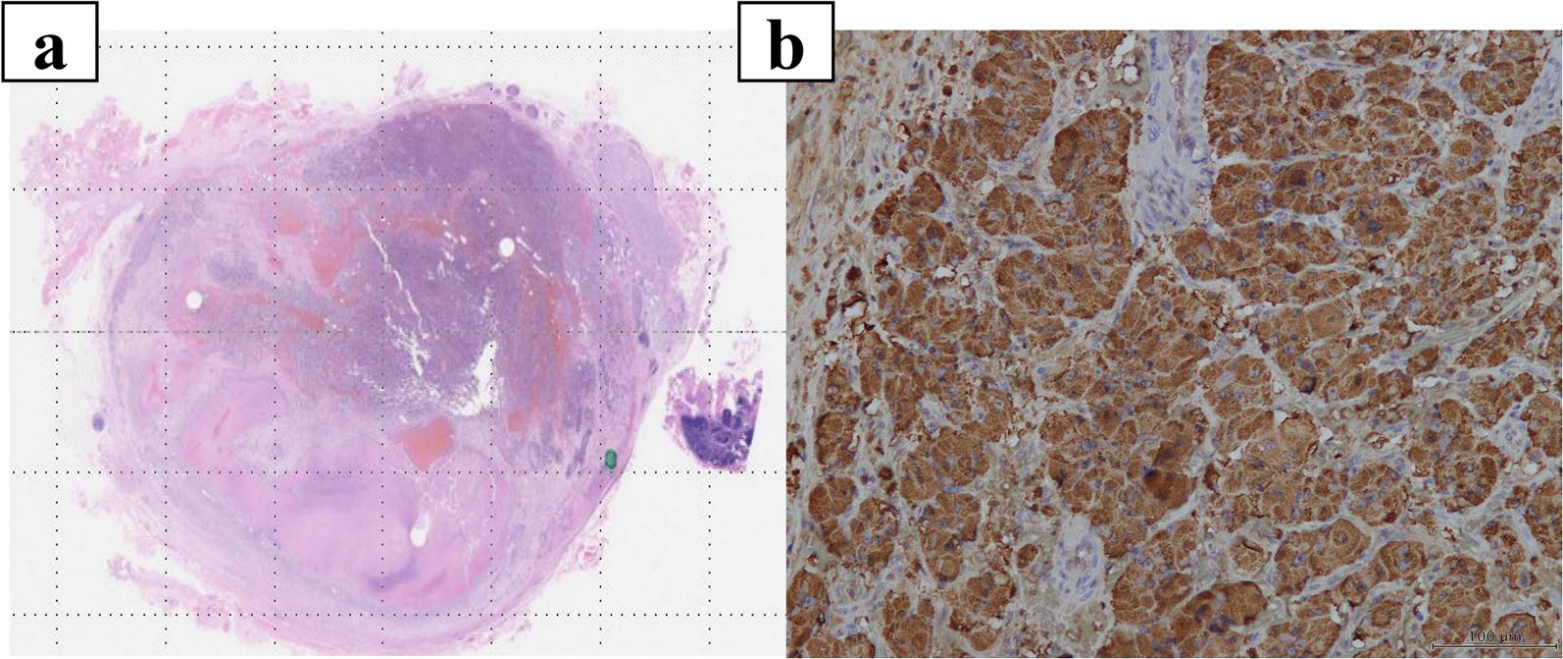
Pathological findings of resected tumor. **a** Pathological examination revealed massive necrosis in the tumor. **b** Immunohistochemistry staining for chromogranin A was broadly positive, leading to a diagnosis of paraganglioma for the resected tumor

A 65-year-old woman came to our hospital for a thorough examination of her hypertension. Blood tests revealed abnormally high levels of adrenaline (0.31 ng/mL), noradrenaline (1.13 ng/mL), and dopamine (0.09 ng/mL). 24-h urinalysis revealed elevated levels of metanephrine (0.35 μg/mg Cre) and normetanephrine (0.22 μg/mg Cre). Computed tomography (CT) detected a 3-cm solid mass located in the dorsal position of the inferior vena cava (Fig. 1a, b). 123I-metaiodobenzylguanidine (MIBG) scintigraphy detected tumor uptake (Fig. 1c). She was diagnosed with functional PGL and started to treat with antihypertensive therapy by alpha1 selective blocker to reduce the risk of sudden perioperative blood pressure changes for elective surgery. Unfortunately, she presented to the emergency department with a chief complaint of abdominal pain on the 56 days after initial diagnosis. On admission, she was hemodynamically stable, with a heart rate of 76 bpm and blood pressure of 161/87 mmHg. Laboratory tests performed in the emergency room revealed a white blood count of 11560/mL, a hemoglobin level of 15.8 g/dL, and normal hepatic enzyme levels. CT showed an enlarged tumor and low-density lesions with fluid retention suggestive of retroperitoneal hemorrhage (Fig. 2a). Angiography showed contrast leakage, which was diagnosed as a retroperitoneal hemorrhage and suspected PGL (Fig. 2b). Considering the risk of a hypercatecholamine crisis, we decided to perform urgent TAE under strict monitoring by an anesthesiologist. The tumor blood flow was supplied from the right adrenal artery and right inferior transverse artery, which was detected by CT at diagnosis (Fig. 3a, b). Subsequently, TAE was selectively performed on these arteries using gelatin sponges. We did not observe any further tumor vascularity on subsequent contrast imaging. After TAE, the patient did not show progressive anemia, and her hemodynamics were stable. In addition, the serum level of catecholamines, such as adrenaline (from 6.27 ng/mL to 0.08 ng/mL), noradrenaline (from 24.88 ng/mL to 0.66 ng/mL), dopamine (from 0.16 ng/ mL to below reference, Fig. 4) were normalized. Three days after TAE, CT revealed a resolving retroperitoneal hematoma. She received alpha 1 selective blocker based antihypertensive therapy to lower blood pressure, and low-dose beta adrenergic receptor antagonist blockers for heart rate control. On the 13 days after TAE, the patient underwent elective open tumor resection, and no significant intraoperative bleeding or blood pressure changes were observed. The operative time was 137 min, with 20 g of blood loss. The gross appearance of the resected tumor was 35 × 30 × 15 mm. The resected tumor was pathologically diagnosed as PGL based on positive immunohistochemical staining results for chromogranin A suggesting massive necrosis (Fig. 5a, b). The Grading of Adrenal Pheochromocytoma and Paraganglioma (GAPP) score was three (cellularity: moderate, one point; Ki67 index, 26%, two-point), indicating moderate malignant potential. Postoperatively, no blood pressure fluctuations or elevated catecholamine levels were recorded. The patient was discharged on postoperative day nine.

## Discussion

Tumor rupture is a rare complication of PPGL that reported as 3.8–24.5% in previous reports [6–8]. It profoundly affects the circulatory system and can be potentially lethal [1]. The mortality rate associated with ruptured PCC is approximately 30% [4]. The mechanism of PPGL rupture is unclear, and rupture could be an early manifestation, even in nonfunctional PPGL [5]. Elective surgeries for ruptured functional PCC after successful antihypertensive therapy reduce associated mortality rates when compared to emergency surgeries [4]. Furthermore, TAE has recently been reported as a suitable option for ruptured PCC to achieve hemodynamic stabilization and permit elective surgery [9]. In 1978, Bunuan et al. first reported that surgical resection 24 h after TAE successfully removed infarcted PCC without hemodynamic incidents [10]. Edo et al. reported that in 74 cases of PCC rupture, the mortality rate of patients who underwent emergency surgery was approximately 40%. There was no mortality in the six patients who underwent elective surgery after TAE [9]. Kobayashi et al. also recommend elective surgery for hemorrhagic PCC after stabilization of circulatory dynamics using TAE [4].

Intraoperative manipulation of a PPGL has been found to result in measurable catecholamine release and significant hemodynamic perturbations [11, 12]. TAE rapidly reduces blood flow to the PCC and inhibits catecholamine secretion, which may lead to rapid stabilization of blood pressure and symptom relief [4]. Previously, one case of TAE treatment was reported for an unruptured retroperitoneal PGL with blood pressure fluctuations, abdominal pain, and hypercatecholaminuria, indicating a hypercatecholamine crisis. TAE of the left inferior phrenic artery successfully relieved abdominal pain and returned serum catecholamine levels to the normal range before elective surgical resection [13]. Although this is the only reported case of TAE treatment for PGL, TAE also worked efficiently in reducing catecholamine levels and perioperative management in our own case. 9 of 10 reported cases including our own case in which surgery was performed after TAE for spontaneous rupture of PPGL successfully completed surgical resection of tumor (Table 1). The dead case was unable to control bleeding either TAE or following operation resulted severe condition due to large amount of blood loss. All the data available cases were normalized their elevation of serum catecholamine level after TAE before surgery. Taken together with our case, TAE could be an option for controlling catecholamine levels in functional PGL. Because PGL arises from an uncertain location, the indication of TAE for PGL depends on the vascular supply. Emergency TAE under close monitoring is an important option for PGL cases with persistent hemorrhage due to tumor rupture [20]. Although further accumulation of cases is needed, TAE would be a potential preconditioning even for the unruptured cases to normalize catecholamine levels for safer intraoperative management.

**Table 1:** Summary of reported PPGL cases treated by TAE following operation

Author/ year	Age/gender	Tumor type	Diameter (cm)	Location	Function	Catecholamine levels	Embolus	Interval for operation	Outcome
Ito K et al./1997 [14]	68/F	PCC	3.0	Lt. AG	N.D	Normalized	PVA	3 months	Alive
Park JH et al./2003 [15]	32/M	PCC	3.8	Rt. AG	Functional	Normalized	Coil	21 days	Alive
Pua U et al./2008 [16]	67/M	PCC	N.D	Rt. AG	Functional	Normalized	PVA	2 months	Alive
Habib M et al./2010 [17]	42/M	PCC	N.D	Rt. AG	N.D	N.D	Coil	1 months	Alive
Hanna JS et al./2010 [8]	38/M	PCC	N.D	Lt.AG	Functional	N.D	Gelatin	4.5 months	Alive
Kumar S et al./2013 [18]	63/M	PCC	N.D	Lt.AG	N.D	N.D	Coil	1 months	Alive
Mukai S et al./2013 [19]	40/M	PCC	10.0	Lt.AG	N.D	N.D	Gelatin	2 h	Died (POD 6)
Edo N et al. /2018 [9]	45/M	PCC	6.5	Lt. AG	Functional	Normalized	N.D.	4.5 months	Alive
Toshiya K et al./2021 [13]	44/M	PGL	4.0	left RP	Functional	Normalized	Gelatin	24 days	Alive
Masataka N et al./2024	65/W	PGL	3.0	RP behind IVC	Functional	Normalized	Gelatin	13 days	Alive

PCC pheochromocytoma, PGL paraganglioma, N.D. no data available, AG adrenal gland, RP retroperitoneum, PVA polyvinyl alcohol

## Conclusion

We report the successful management of a patient who underwent elective surgery after TAE for spontaneous PGL rupture. TAE for PGL could be a useful option functional PGL.

## Data Availability

Data sharing is not applicable to this article, as no datasets were generated or analyzed during the current study.

## Abbreviations

CT: Computed tomography
PGL: Paraganglioma
PCC: Pheochromocytoma
PPGL: Pheochromocytomas and paragangliomas
TAE: Transcatheter arterial embolization
GAPP: Grading of Adrenal Pheochromocytoma and Paraganglioma
